# Impact of Processing on the Phenolic Content and Antioxidant Activity of *Sorghum bicolor* L. Moench

**DOI:** 10.3390/molecules29153626

**Published:** 2024-07-31

**Authors:** Aduba Collins, Abishek Santhakumar, Sajid Latif, Kenneth Chinkwo, Nidhish Francis, Christopher Blanchard

**Affiliations:** 1 School of Dentistry and Medical Sciences, Faculty of Science and Health, Charles Sturt University, Wagga Wagga, NSW 2650, Australia; njok@csu.edu.au (A.C.); kchinkwo@csu.edu.au (K.C.); cblanchard@csu.edu.au (C.B.); 2 Gulbali Institute, Charles Sturt University, Wagga Wagga, NSW 2650, Australia; nfrancis@csu.edu.au; 3 National Life Sciences Hub, Charles Sturt University, Wagga Wagga, NSW 2650, Australia; slatif@csu.edu.au; 4 School of Agricultural, Environmental and Veterinary Sciences, Charles Sturt University, Wagga Wagga, NSW 2650, Australia

**Keywords:** polyphenols, sorghum, fermentation, cooking, antioxidant

## Abstract

Sorghum, a cereal grain rich in nutrients, is a major source of phenolic compounds that can be altered by different processes, thereby modulating their phenolic content and antioxidant properties. Previous studies have characterised phenolic compounds from pigmented and non-pigmented varieties. However, the impact of processing via the cooking and fermentation of these varieties remains unknown. Wholegrain flour samples of Liberty (WhiteLi_1_ and WhiteLi_2_), Bazley (RedBa_1_ and RedBa_2_), Buster (RedBu_1_ and RedBu_2_), Shawaya black (BlackSb), and Shawaya short black 1 (BlackSs) were cooked, fermented, or both then extracted using acidified acetone. The polyphenol profiles were analysed using a UHPLC-Online ABTS and QTOF LC-MS system. The results demonstrated that combining the fermentation and cooking of the BlackSs and BlackSb varieties led to a significant increase (*p* < 0.05) in total phenolic content (TPC) and antioxidant activities, as determined through DPPH, FRAP, and ABTS assays. The 2,2′-azino-bis (3-ethylbenzothiazoline-6-sulfonic acid) (ABTS) radical scavenging activity of WhiteLi_1_, BlackSb, RedBu_2_, and BlackSs increased by 46%, 32%, 25%, and 10%, respectively, post fermentation and cooking. Conversely, fermentation only or cooking generally resulted in lower phenolic content and antioxidant levels than when samples were fully processed compared to raw. Notably, most of the detected antioxidant peaks (53 phenolic compounds) were only detected in fermented and cooked black and red pericarp varieties. The phenolic compounds with the highest antioxidant activities in pigmented sorghum included 3-aminobenzoic acid, 4-acetylburtyic acid, malic acid, caffeic acid, and luteolin derivative. Furthermore, the growing location of Bellata, NSW, showed more detectable phenolic compounds following processing compared to Croppa Creek, NSW. This study demonstrates that sorghum processing releases previously inaccessible polyphenols, making them available for human consumption and potentially providing added health-promoting properties.

## 1. Introduction

Increased awareness of the health-promoting benefits of wholegrains has prompted investigations into the benefits of processing for dietary consumption [1,2]. Sorghum whole grains are rich in health-promoting phenolic compounds such as phenolic acids, flavonoids, and anthocyanins, primarily found in varieties with darker pigmentation [3,4]. These compounds possess antioxidant and free radical scavenging properties that have been shown to exhibit a protective role against oxidative stress, inflammation, obesity, and cancer development [5,6,7].

Processing technologies have primarily focused on reducing the anti-nutritional content in sorghum grains. However, food processing techniques have the potential to transform raw materials into edible and nutritious food [8]. Most wholegrains are not consumed raw but go through industrial or domestic processing, impacting their taste, composition, and bioaccessibility. Some of the common food processes include the open-pan boiling, fermentation, and cooking of fermented flour [2]. For example, sorghum porridge, known as “medida” in east African countries, is boiled from a slurry of milled flour [9]. The preliminary step of fermentation changes the porridge’s smell, texture, taste, and phenolic antioxidants [10]. This is due to microbial and enzymatic activity during fermentation that causes the release of bound polyphenols, resulting in variations to the antioxidant activities of sorghum metabolites [11]. 

Variations in the phenolic profile and antioxidant activities in processed sorghum can also be due to the growing location, processing time, the presence of microbes, and their enzymatic activity [12]. During fermentation, microorganisms, such as *Lactobacillus* bacteria, can alter the composition of phenolic compounds by producing polyphenol-associated enzymes [13]. These enzymes have been reported to release bound phenols, e.g., tannates, esterases, phenolic acid decarboxylates, and glucosidases [14]. Moreover, cooking can result in the loss of crystalline structures in the starch molecules amylose and amylopectin [15]. As a result, more water is absorbed by the starch molecules, leading to interactions with proteins and polysaccharides and, ultimately, the formation of highly accessible and bioactive water-soluble compounds. 

It is crucial to optimise sorghum cooking to improve its nutritional content, mainly phenolic compounds associated with disease prevention. An extensive amount of research has primarily focused on raw sorghum grain polyphenols, with emerging studies on the impact of different processes [16,17]. The findings of this study provide novel insights into the changes in phenolic composition and antioxidant activity of sorghum grain polyphenols after processing via cooking, fermentation, and a combination of both. Furthermore, an appropriate selection of sorghum genotypes and processing methods that inform the consumer market for foods with notable health benefits is warranted. Therefore, this study aimed to determine the effect of various cooking and processing techniques on the phenolic content and antioxidant activity of eight pigmented and non-pigmented sorghum varieties. A UHPLC-online ABTS system coupled with QTOF LC-MS was also used to identify and quantify phenolic compounds in the processed and unprocessed sorghum varieties. This study provides a comprehensive understanding of the impact of various sorghum cooking and preparation techniques for optimum use for human consumption with added health benefits.

## 2. Results and Discussion

### 2.1. Impact of Processing on Total Phenolic Content (TPC)

**Figure 1:**
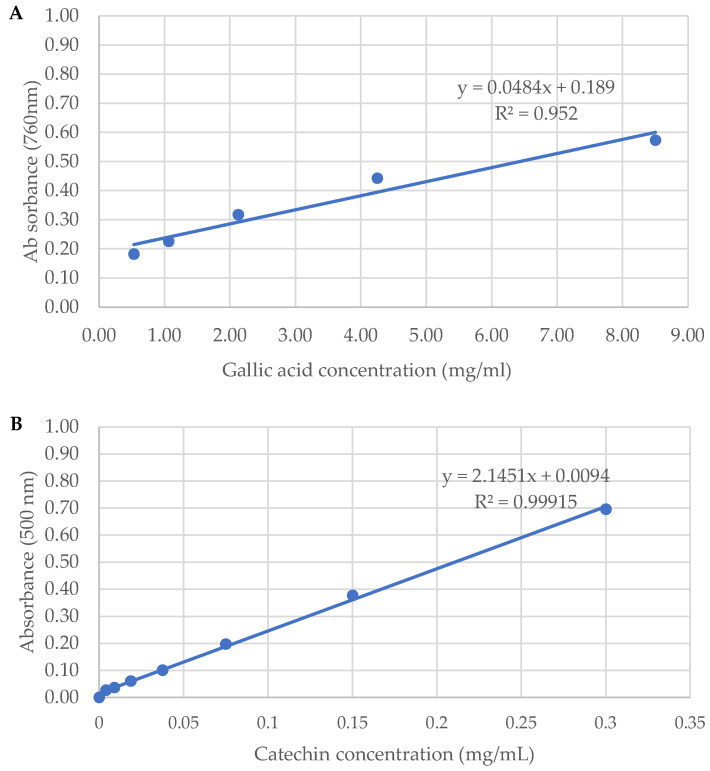
Standard calibration curves for the determination of (**A**) total phenolic content and (**B**) total proanthocyanidin content (TPAC).

The eight sorghum varieties investigated exhibited a significant variation in TPC when compared to a gallic acid standard curve *p <* 0.05 (Table 1, Figure 1). Raw BlackSs had the highest TPC at 9.66 ± 1.66 mg/g GAE. An increase in TPC to 10.93 ± 0.85 mg/g GAE was observed post fermentation and cooking. A similar trend was observed in BlackSb, which had the second highest TPC at 6.67 ± 0.64 mg/g GAE in raw extracts. The TPC of BlackSb also increased to 6.86 ± 0.73 mg/g GAE following fermentation and cooking (Table 1). In comparison, the TPC of RedBa_2_ and WhiteLi_1_ did not change after fermentation and cooking (0.56 ± 0.04 mg/g GAE to 0.55 ± 0.12 mg/g GAE and 0.33 ± 0.01 mg/g GAE to 0.34 ± 0.11, respectively) (Table 1). The fermentation process increased the TPC of BlackSb, RedBa_1_, and WhiteLi_1_ (Table 1). Cooking decreased the TPC for all pigmented varieties except for RedBa_1_ (0.50 ± 0.13 mg/g GAE to 0.67 ± 0.25 mg/g GAE). These TPC findings suggest that sorghum processing causes a breakdown in the structural characteristics of some phenolic compounds, thereby increasing their accessibility for detection [18]. The mechanism of action during the cooking process is likely due to the starch content in sorghum grains expanding, becoming compressed with increasing temperatures and producing amylose [15]. Additionally, part of the starch granules can then form starch–lipid complexes that can interfere with the hydrolysis of bioactive compounds, explaining the decrease in TPC [14,19]. However, during the fermentation process, live microorganisms such as *Lactobacillus* bacteria are introduced [13]. These bacteria can result in the biotransformation of polyphenols and the production of enzymes that release bound phenols [16]. Furthermore, it is evident that the combined cooking and fermentation processes had the most favourable outcome due to the release of the bound polyphenols from starch molecules and pH modifications after fermentation [20].

The variation in the TPC of sorghum primarily depends on pericarp pigmentation, genotype, and, as previously demonstrated, susceptibility to environmental conditions at different growing locations [21]. In addition, extraction methods using solvents such as ethanol or hexane also contribute to variations in TPC levels [22,23]). A study by Wu et al. (2017) [24] used an 80% aqueous methanol extraction method to obtain free phenolics. In comparison, the current study used an acidified acetone extraction method (70% acetone, 29.5% water, and 0.5% acetic acid) and found raw TPC levels to be slightly lower for BlackSs. However, the TPC of fermented cooked BlackSs increased, demonstrating that the fermentation and cooking processes have a greater impact on phenolic concentrations despite the extraction solvents used.

### 2.2. Total Proanthocyanidin Content (TPAC)

Commonly known as proanthocyanidins, tannins and condensed tannins have been reported in darker-pigmented sorghum varieties in high quantities [25,26]). The current study determined that the fermented and cooked processes increased the TPAC for all varieties when compared to a catechin standard curve (*p* < 0.05) except for BlackSs when compared to raw samples (8.38 ± 0.44 mg/g CE to 7.05 ± 0.58 mg/g CE) (Table 1, Figure 1). Despite cooking decreasing the TPAC of BlackSs by 50% (8.38 ± 0.44 mg/g CE to 4.88 ± 0.67 mg/g CE), the fermentation process increased the cooked TPAC of BlackSs (4.88 ± 0.67 mg/g CE, 6.44 ± 0.03 mg/g CE and 7.05 ± 0.58 mg/g CE, respectively). Interestingly, RedBa_2_ and RedBu_2_ showed higher TPAC concentrations for the fermented cooked processes than RedBa_1_ and RedBu_1_. The variations in TPAC are presumably due to the release and detection of cell-wall-bound proanthocyanidins, leading to the synthesis of free forms of various bioactive compounds [27]. A study demonstrated that red sorghum processing decreased TPAC by 64% then increased it by 52% depending on the technique used [28]. The degradation of proanthocyanidin oligomeric species into smaller and more-soluble monomeric and dimeric units was determined as the mechanism of action. Susanti et al. (2021) [17] also determined that processing red sorghum via extract encapsulation increased the proanthocyanidin content from 9.11 mg/g to 9.23 mg/g. Therefore, the changes to TPAC as a result of processing in the current study agree with the range of values described in the literature.

### 2.3. Post Processing Variation in Antioxidant Activity

Three antioxidant assays, including ABTS, DPPH, and FRAP, were used to determine the overall antioxidant and radical scavenging activity of eight sorghum varieties. Fermented and cooked sorghum samples exhibited a significant increase in ABTS for all eight varieties (*p* < 0.05). WhiteLi_1_ showed a two-fold increase in antioxidant activity (0.25 ± 0.05 mg 100^−1^ TE to 0.54 ± 0.07 mg 100^−1^ TE) followed by BlackSb (32%) and RedBu_2_ (25%) after combining fermentation and cooking (Table 1). Cooking also increased the antioxidant activity of all varieties; however, BlackSs showed a 30% decrease in ABTS. A possible explanation for the increased ABTS is that darker-pigmented varieties of sorghum lack or contain low levels of the starch components amylose and amylopectin, which can be attached to bound polyphenols. A study reported that the starch content of sorghum varies greatly between 32.1 and 72.5 g/100 g [29]. Rather than releasing the antioxidants, the high temperatures during cooking may degrade, causing a loss of water-soluble compounds [13]. The production of melanoidins via the “Maillard reaction” has also been reported as a by-product of amino acids reacting with sugars when food is cooked at high temperatures [30]. These compounds can decrease phenolic recovery due to heat-induced degradation of flavonols. Interestingly, BlackSb did not exhibit a decrease in antioxidant activity, demonstrating that this sorghum variety may contain compounds with structural bonds that can withstand high temperatures during cooking.

The DPPH results demonstrated significant differences in antioxidant activity (*p* < 0.05). Similar to the ABTS, six of the eight sorghum varieties demonstrated a surge in DPPH following fermentation and cooking (Table 1). Despite the two red varieties, RedBa_1_ and RedBu_1_, exhibiting a decrease in DPPH when fermented and cooked, their antioxidant activities increased when compared to fermentation alone (1.99 ± 0.17 mg/g TE to 2.33 ± 0.12 mg/g TE and 1.80 ± 0.17 mg/g TE to 2.15 ± 0.15 mg/g TE, respectively). It is reasonable to assume that the cultivation location (Bellata, NSW) influenced the DPPH of the detectable phenolic compounds despite the implementation of the different processes used. Bianco-Gomes et al. (2022) [31] and Adebo et al. (2018) [32] have reported similar results of heat-processing and fermentation, causing an increase in radical scavenging activity. These processes induce the liberation of structurally bound phenolic compounds in addition to hydrolysis and depressurisation at the end of the heating process. 

The FRAP radical scavenging activity of the processed sorghum varieties was determined by their ability to reduce Fe (III). The fermented cooked process exhibited the greatest radical scavenging activity in seven sorghum varieties (*p* < 0.05) (Table 1). BlackSb, RedBu_1_, WhiteLi_1_, and WhiteLi_2_ showed the highest antioxidant activity, increasing by 40–60% when fermented and cooked. RedBa_2_ remained unchanged at FRAP values of 5.56 ± 0.04 mg/g TE to 5.53 ± 0.65 mg/g TE and decreased when fermented only (5.56 ± 0.04 mg/g TE to 4.55 ± 0.72 mg/g TE); however, this was not a significant variation. Fermentation alone also significantly decreased FRAP in BlackSs by 32%, demonstrating that the altered pH from the resultant acid production may cause a loss in detectable phenolic compounds. Unexpectedly, ferric reducing ability was increased when cooking alone was implemented for all eight varieties, with the highest FRAP value found for BlackSs (23.27 ± 3.50 mg/g TE to 28.57 ± 2.22 mg/g TE), followed by BlackSb (7.29 ± 1.26 mg/g TE to 9.13 ± 1.55 mg/g TE) and RedBa_1_ (6.69 ± 0.66 mg/g TE to 8.24 ± 1.46 mg/g TE). Zaroug et al. (2014) [33] reported an increase in FRAP radical scavenging activity after red pericarp sorghum dough baking but varied results after fermentation for 24 h and then 32 h (gradual increase, then a 45% decrease). A recent study also found similar FRAP results for processed pigmented red and non-pigmented white pericarp sorghum varieties [28]. The results in the current study differed from the mentioned studies as the processing technologies implemented increased the antioxidant and radical scavenging activity of all eight varieties, especially the black varieties. This suggests that the influence of domestic cooking techniques exhibits favourable outcomes in some sorghum varieties over others. Similar to the other assays, we hypothesise that the disruption to the cell wall matrix of high-molecular-weight compounds provides phenolic accessibility for extraction to occur, revealing highly detectable compounds [34].

### 2.4. Quantification and Identification Using UHPLC with Online ABTS and QTOF LC-MS

**Figure 2:**
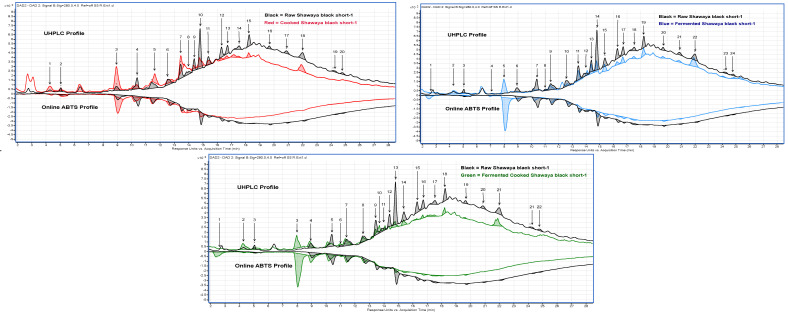
UHPLC—Online ABTS overlaid changes in phenolic compounds after processing from black pericarp sorghum variety Shawaya short black 1 (BlackSs). * Compound detected in multiple processes. The numbers represent compounds identified in each process for BlackSs.

**Figure 3:**
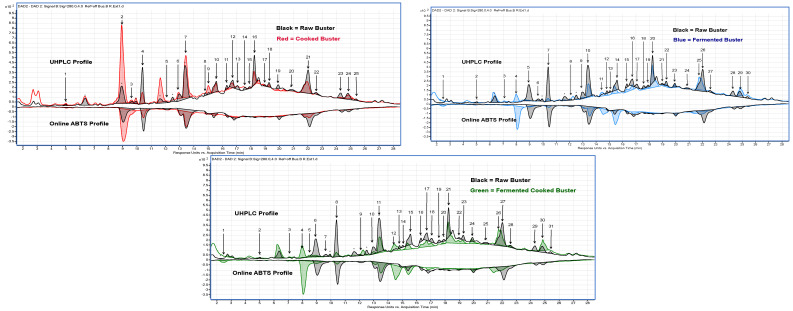
UHPLC—Online ABTS overlaid changes in phenolic compounds after processing from red pericarp sorghum variety Buster (RedBu_1_). * Compound detected in multiple processes. The numbers represent compounds identified in each process for RedBu_1_.

**Figure 4:**
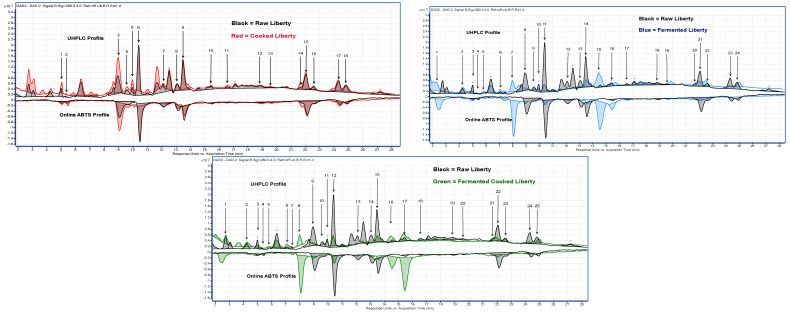
UHPLC—Online ABTS overlaid changes in phenolic compounds after processing from white pericarp sorghum variety Liberty (WhiteLi_1_). * Compound detected in multiple processes. The numbers represent compounds identified in each process for WhiteLi_1_.

**Figure 5:**
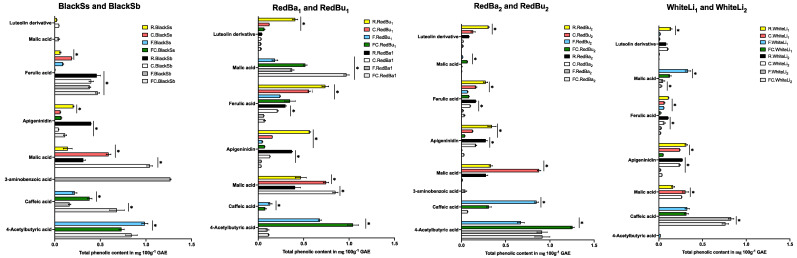
Variations in the phenolic composition (determined by a gallic acid standard curve in a UHPLC—Online ABTS system) of the significant phenolic compounds identified before and after processing of sorghum samples. Data are expressed as mg 100^−1^ GAE and presented as mean ± SD; *n* = 3. Two—way ANOVA with multiple comparisons was performed, and statistical significance was set at *p* ≤ 0.05. * Statistically significant difference within processed sorghum samples. BlackSs, Shawaya short black 1; BlackSb, Shawaya black; RedBa_1_ and RedBa_2_, Bazley; RedBu_1_ and RedBu_2_, Buster; WhiteLi1 and WhiteLi2, Liberty; R, raw; C, cooked, F, fermented; FC, fermented and cooked.

The UHPLC-online ABTS characterisation of the eight sorghum varieties exhibited a significant variation in total phenolic content and antioxidant activity (*p* < 0.05). Cooking, fermentation, and a combination of both processes showed a higher number of different phenolic compounds in the black sorghum varieties than in red and white. A total of 87 peaks were identified (Table 2 and Figure 2, Figure 3 and Figure 4) across the eight varieties with measurable antioxidant activity. Out of these, only seven phenolic compounds were retained from the raw extracts and throughout the fermentation and cooking processes. These compounds were trans-resveratroloside, catechin, glucomalcomiin, luteolin-7-*O*-glucoside, taxifolin, voacamine, and taxifolin-*O*-pentoside. Most of the peaks (53 phenolic compounds) were detected only after processing either with cooking, fermentation, or both. The compound 3-aminobenzoic acid (peak 22 and 56) was the major polyphenol detected following fermentation. This compound was detected in fermented or fermented cooked RedBa_2_, RedBu_1_, and WhiteLi_1_ and with abundance in the BlackSb variety at 1.27 ± 0.01 mg 100 g^−1^ GAE (Figure 5). The phenolic compound 4-acetylbutyric acid (peak 20) was primarily detected in fermented cooked samples and was abundant in fermented cooked RedBu_2_. The TPC of this compound increased in RedBu_1_ and RedBu_2_ by two-fold from 0.68 ± 0.02 to 1.04 ± 0.06 and 0.67 ± 0.04 to 1.26 ± 0.02 mg 100 g^−1^ GAE, which correlates with the benchtop TPC results in Table 1. Ferulic acid (peak 43) was the major compound in all the raw sorghum samples (Figure 5). This compound was detected in all sorghum varieties for all processing techniques, with the most abundant amount detected in raw RedBu_1_ and fermented cooked BlackSb at 0.74 ± 0.04 and 0.47 ± 0.02 mg 100 g^−1^ GAE. Eighteen compounds were detected in all sorghum varieties, including apigeninidin (peak 31) and n’.n’-dicafferoylspermidine (peak 42). However, several processed samples of BlackSs, BlackSb, RedBu_2_, RedBa_2_, and WhiteLi_1_ did not have detectable amounts of these compounds. Caffeic acid (peak 21) was the most abundant in fermented RedBu_2_ at 0.85 ± 0.02 followed by fermented and fermented cooked WhiteLi_2_ at 0.83 ± 0.03 and 0.76 ± 0.04 mg 100 g^−1^ GAE, respectively. Malic acid (peak 24 and 46) was the major compound in cooked sorghum samples; it was abundant in cooked BlackSb at 1.04 ± 0.03 mg 100 g^−1^ GAE and, secondly, in fermented cooked RedBa_1_ at 0.97 ± 0.03 mg 100 g^−1^ GAE. Only four compounds were identified exclusively after cooking the black and red sorghum varieties—vanillin, luteolin derivative, eriodictyol deoxyhexoside, and apigenin.

**Figure 6:**
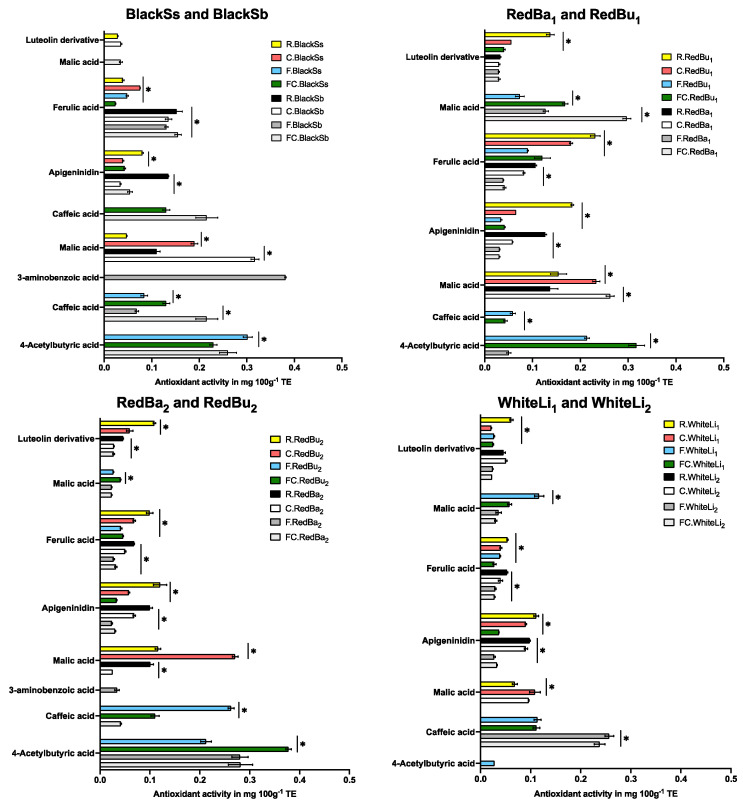
Changes to the ABTS antioxidant activity (determined by a Trolox standard curve in a UHPLC—Online ABTS system) of the significant phenolic compounds identified before and after processing of sorghum samples. Data are expressed as mg 100^−1^ TE and presented as mean ± SD; *n* = 3. Two—way ANOVA with multiple comparisons was performed, and statistical significance was set at *p* ≤ 0.05. * Statistically significant difference within processed sorghum samples. BlackSs, Shawaya short black 1; BlackSb, Shawaya black; RedBa_1_ and RedBa_2_, Bazley; RedBu_1_ and RedBu_2_, Buster; WhiteLi_1_ and WhiteLi_2_, Liberty; R, raw; C, cooked, F, fermented; FC, fermented and cooked.

The numbers in the level of ID column indicate the source of tentatively identified compounds. The number 1 indicates that the compound was identified using a standard; 2 indicates that the compound was identified using Massbank (Massbank, 2006) database; 3 indicates that the compound was identified using the Chemspider (Chemspider, 2024) database; 4 indicates that the compound was identified using the PubChem (PubChem, 2024) database; 5 indicates that the compound was identified using the Metlin (Metlin, 2006) database.Antioxidant activity was higher in processed sorghum extracts compared to raw (Figure 6). The highest antioxidant activity was exhibited by 3-aminobenzoic acid (peak 22) and 4-acetylbutyric acid (peak 20) in fermented BlackSb and fermented cooked RedBu_2_ at 0.38 ± 00 and 0.32 ± 0.02 mg 100 g^−1^ TE, respectively. Luteolin derivative (peak 80) reduced in antioxidant activity following processing in all varieties except for RedBa_1_ and WhiteLi_2_, which did not change. The antioxidant activity of caffeic acid (peak 21) in RedBa_2_ and WhiteLi_2_ also did not change following fermentation and cooking. In the varieties BlackSb, RedBa_2_, RedBu_1_, and WhiteLi_1_, the concentration of 3-aminobenzoic acid (peak 56) increased, which resulted in a decrease in malic acid (peak 46). 

These phenolic compounds displayed opposite peak sizes when samples were fermented and then cooked (Figure 3 and Figure 4), suggesting that processing influences certain compounds’ free radical scavenging activity in connection to others. Generally, cooking causes a reduction in the phenolic content and antioxidants. However, the fermentation step generally resulted in increased, or retention of, total phenolic content and the antioxidant activity of some sorghum polyphenols. Several compounds were identified only due to the application of cooking or fermentation or both. 

Similar to other cereal processes, fermentation of sorghum flour leads to a modification in integral metabolites, a decrease in pH, increased microbial activity, and the activation of enzymes [11]. These enzymes can hydrolyse the β-glucosidic bonds of various phenolic compounds present as conjugates with sugar residues linked to hydroxyl groups [18]. The proposed mechanism of action is the breakdown of the cell wall matrix by the secretions of fermenting microbes. Furthermore, it can be hypothesised that the microbial action by *Lactobacillus* strains and other aerobic microflora during fermentation led to the changes in antioxidant activities in our study [16]. Additionally, it has been reported that shelf life, aroma, and structural changes are also apparent due to fermentation [18]. In the current study, the flour samples contained distinct differences following processing, such as a finer texture, colour change, and a noticeable scent. Investigations by Zaroug et al. (2014) [33] used 8, 16, 24, and 32 h fermentation time points and determined that total phenol, tannins, and flavonoids content increased with longer fermentation periods. This observation agrees with the current study, which used 48 h as the maximum fermentation time. 

The total phenolic content of the eight raw sorghum varieties observed in this study was similar to the values reported in the literature [16,35]. Our study detected a greater number of different polyphenols scarcely identified in unprocessed sorghum grains. These include stilbenes, such as trans-piceid and trans-resveratroloside, which were detected in RedBa_1_, RedBu_1_, and RedBu_2_. Also, catechin and its isomers were only detected in BlackSb and BlackSs. The current study observed the radical scavenging activity in the black sorghum varieties, predominantly attributed to various concentration changes for malic acid, 3-aminobenzoic acid, and 4-acetylbutyric acid. In contrast, Punia et al. (2021) [27] observed higher antioxidant activities for caffeic acid, taxifolin, and apigeninidin, which were also identified in the current study. However, these compounds were not as greatly affected by processing. 

It has been reported that food processing depends upon factors such as cultivation conditions and the genotype of the grain [8]. The composition of polyphenols varied not only between the processes implemented but also between the same varieties from different growing locations. Three major differences were observed: the detection of gallic acid (peak 11) in only one white variety (WhiteLi_1_), linoleic acid (peak 15) in one red variety (RedBa_1_), and epicat-(4beta → 6)-epicatechin-(2beta → 7,4beta → 8)-epicatechin (peak 58) in one red variety (RedBu_1_), all of which were grown in Bellata, NSW. Therefore, the growing location could have a major impact on the different compounds present in grain samples and their potential to be released after food processing. Interestingly, the polyphenols detected in each sorghum sample varied in number with respect to the processing technique, e.g., raw, cooked, fermented, and fermented cooked BlackSs had 22, 23, 16, and 21 compounds, respectively. A similar trend was observed for other varieties; however, the antioxidant concentrations generally increased. As previously mentioned, a possible explanation is the breakdown of polymers and covalent bonds, allowing for the observation of otherwise undetectable polyphenols.

Food processing has had a negative reputation for reducing the nutritional benefits of functional foods [9]. However, cooked sorghum, with or without the fermentation step, has the potential to be introduced into the human diet due to its diverse range of bioactive compounds. Potential health benefits are attributed to several of the detected flavonoid compounds identified in this study. For example, apigeninidin and ferulic acid have been attributed to reducing oxidative stress and the onset and development of gastrointestinal cancers [7]. In this study, we have demonstrated that the fermentation and cooking processes can provide higher accessibility to compounds with known health benefits in sorghum grains. In contrast to the study by Hithamani and Srinivasan (2014) [9], the current study showed that the concentration of ferulic acid was retained following cooking, fermentation, and a combination of both. This compound has been reported to reduce tumour-promoting inflammatory markers and decrease cancer cell growth in vivo [35,36]. Several compounds that were identified have yet to be studied for their biological activity, particularly those detected only after one or more processes were applied to the sorghum samples. 

## 3. Materials and Methods

### 3.1. Materials

#### 3.1.1. Samples

Eight different pigmented sorghum (*Sorghum bicolor* L. Moench) grains were used for experimentation. One Liberty white and two Bazley and Buster red varieties (WhiteLi_1_, RedBa_1_, RedBu_1_) were grown in 2021 in Bellata, New South Wales, as part of the New South Wales Department of Primary Industries (DPI) field trials. Identical varieties were grown in 2021 in Croppa Creek, New South Wales (WhiteLi_2_, RedBa_2_, RedBu_2_). The black varieties Shawaya short black-1 and Shawaya black (BlackSs and BlackSb) were grown in 2021 glasshouse trials conducted at the Hermitage Research Facility, Warwick, Queensland, by the Department of Agriculture and Fisheries. All sorghum samples were collected at maturity and stored in cold rooms at 4 °C. Experimental analysis for biological and technical replicates was conducted in triplicate. 

#### 3.1.2. Standards and Reagents

Chemicals were purchased from Chem Supply Pty Ltd. (Port Adelaide, SA, Australia), ThermoFisher (Scoresby, VIC, Australia), or Sigma-Aldrich (St. Louis, MO, USA). 

Hexane, acetone, acetic acid, methanol, sodium carbonate, potassium persulfate, hydrochloric acid, and sulphuric acid were obtained from Chem Supply Pty Ltd. Trolox (6-Hydroxy-2,5,7,8-tetra-methylchroman-2-carboxylic acid), DPPH (2,2-diphenyl-1-picrylhydrazyl), Folin–Ciocalteu reagent, TPTZ (2,4,6-tris(2-pyridyl)-s-tria-zine), ABTS (2,2′-azino-bis (3-ethylben-zothiazoline-6-sulfonic acid), iron (III) chloride, formic acid, peonidin 3-*O* glucoside chloride, delphinidin chloride, apigenin, petunidin 3-*O* glucoside, luteolin, procyanidin B3, cyanidin-3-*O* glucoside, procyanidin B1, phosphotungstic acid, isovanillic acid, hippuric acid, gallic acid, protocatechuic acid, syringic acid, vanillic acid, ellagic acid, trans-cinnamic acid, o-coumaric acid, caffeic acid, p-coumaric acid, phlorodizin, sinapic acid, chlorogenic acid, rutin, catechin, naringenin, coumarin, quercetin, ferulic acid, and vanillin were purchased from Sigma-Aldrich.

### 3.2. Methods

#### 3.2.1. Flour Preparation

Sorghum flour was obtained by finely grinding wholegrain sorghum with a 0.5 mm sieve in a Retsch Ultra Centrifugal Mill ZM 200 (Haan, North Rhine-Westphalia, Germany). Sorghum flour was defatted three times with hexane at a ratio of 1:20 (*w*/*v*) and air-dried overnight. Defatted, raw sorghum flour samples were stored at 4 °C for subsequent processing.

#### 3.2.2. Cooking of Sorghum Flour

The cooking method by Proietti et al. (2013) [37] was used to obtain sorghum porridge samples. A slurry was obtained by mixing 10 g of each previously prepared flour sample with 20 mL of sterile water. The slurry was added to 80 mL boiling water with constant stirring for 10 min at approximately 95 °C to obtain porridge then cooled in a water bath for 5 min. Cooled sorghum porridge samples were lyophilised at −80 °C in a Model Alpha 2–4 LD Plus Christ freeze dryer (Biotech International, Kaltenkirchen, Germany), and the resultant dry samples were stored at 4 °C for subsequent analysis. 

#### 3.2.3. Fermentation of Sorghum Flour

The fermentation process method for this study was performed according to traditional methods as commonly practiced in African countries [38]. Briefly, eight varieties of sorghum flour (10 g each) were mixed with 20 mL of cool sterilised water to obtain a slurry. The mixtures were covered, sealed, and left to ferment at room temperature (25 °C) for 48 h with the natural microflora to pH 4. To stop the fermentation process, 80 mL of cool sterilised water was added to each mixture to obtain a ratio of 1:10 (*w*/*v*). The samples were lyophilised at −80 °C in a Model Alpha 2–4 LD Plus Christ freeze dryer (Biotech International, Germany), and the resultant dry samples were stored at 4 °C for subsequent analysis.

#### 3.2.4. Cooking of Fermented Sorghum Flour

To obtain fermented cooked sorghum samples, a combination of the methods by Correia et al. (2010) [38] and Proietti et al. (2013) [37] was used. As mentioned, defatted flour samples were fermented for 48 h at room temperature (25 °C). Following lyophilisation, 10 g of fermented, dry samples was mixed with 20 mL of sterile water, and the slurry was added to 80 mL of boiling water. The samples were lyophilised at −80 °C in a Model Alpha 2–4 LD Plus Christ freeze dryer (Biotech International, Germany), and the resultant dry samples were stored at 4 °C for subsequent analysis. For each sorghum variety, four samples were obtained: R (raw), C (cooked), F (fermented), and FC (fermented cooked). All sample preparations were performed in triplicate.

#### 3.2.5. Polyphenol Extraction

The extraction technique was adopted from earlier studies conducted by our research group [39]. An extraction solvent of acetone, water, and acetic acid solution (70:29.5:0.5 *v*/*v*/*v*) was added at a ratio of 1:10 (*w*/*v*) to the defatted R, C, F, and FC flour samples. The mixture was stirred for 1 h at room temperature (25 °C), and the mixtures were centrifuged at 4000 rpm for 10 min at room temperature and pooled to collect the supernatants. This step was repeated three times to a total volume of 30 mL per gram of sample. Acetone was removed via rotary vacuum evaporation (Rotavapor R-210 BUCHI Labortechnik, Flawil, Switzerland) at 50 °C. The remaining liquid in the samples was then frozen at −80 °C then freeze-dried (Christ-Alpha 2–4 LD Plus freeze dryer, Biotech International, Germany) and stored at −20 °C until further analysis. For analysis, dried sorghum extracts were reconstituted in 50% methanol and later stored at −20 °C. The extraction procedure was performed in triplicate for all samples.

#### 3.2.6. Total Phenolic Content

Total phenolic content was determined via the method described by Noreen et al. (2017) [40] with slight modifications. Briefly, 125 μL of reconstituted sorghum extract was mixed with 125 μL Folin–Ciocalteu reagent and 500 μL of deionised water and incubated in the dark for 6 min. Following incubation, 1.5 mL of 7% sodium carbonate and 1 mL of deionised water were added to neutralise the reaction. After a 90 min incubation period at room temperature in the dark, samples were transferred to 96-well plates and the absorbance was measured at 760 nm using a microplate reader (BMG Labtech Fluostar Omega, Offenburg, Germany). A gallic acid standard curve (Figure 1) was used for the quantification of phenolic content of the samples, and the data were expressed as mg/g gallic acid equivalents (GAE). Analyses were conducted in triplicate for each replicate.

#### 3.2.7. Total Proanthocyanidin Content (TPAC)

The vanillin assay was used for this study as adapted from Min et al. (2011) [41] to quantify proanthocyanidins. A 200 μL aliquot of reconstituted sorghum extract was combined with 500 μL of 1% (*w*/*v*) vanillin in methanol and 500 μL of 25% sulphuric acid in methanol. The mixture was then incubated at 37 °C for 15 min. After incubation, absorbance was measured at 500 nm on a microplate reader (BMG Labtech Fluostar Omega, Offenburg, Germany). Total proanthocyanidins content was quantified using a (+)-catechin standard curve (Figure 1) and the content in the samples was expressed as mg/g (+)-catechin equivalents (CE). Analyses were conducted in triplicate for each replicate.

#### 3.2.8. DPPH Radical Scavenging Activity

The DPPH (1,1-diphenyl-2-picrylhydrazyl, Sigma-Aldrich, Burlington, MA, USA) radical scavenging activity was determined using the method adapted from [42] with slight modifications. Briefly, 200 μL of reconstituted sorghum samples were mixed with 800 mL of 0.2 mM DPPH solution dissolved in 99.9% methanol, left to react for 30 min, and the absorbance was measured at 520 nm (Thermo Scientific Co. Ltd., Waltham, MA, USA). DPPH scavenging activity was expressed in mg of TE per g. A Trolox standard curve was used to quantify DPPH antioxidant activity, and the data were expressed as mg/g Trolox equivalents (TE). Analyses were conducted in triplicate for each replicate.

#### 3.2.9. Ferric Reducing Ability of Plasma (FRAP) Assay

The FRAP assay method described by Sompong et al. (2011) [43] was used with minor modifications. Freshly prepared FRAP reagent consisting of 100 mL acetate buffer (300 mM, pH 3.6), 10 mL FeCl_3_·6H_2_O (20 mM), and 10 mL TPTZ solution (10 mM TPTZ in 40 mM HCl) in a ratio of 10:1:1 was used. A 1.8 mL aliquot of FRAP reagent was combined with 180 μL of deionised water, and 60 μL of reconstituted sorghum extract was then gently vortexed. The mixture was incubated for 40 min at 37 °C. Following incubation, absorbance was recorded at 593 nm using a microplate reader (FLUOstar Omega microplate reader, BMG Labtech, Offenburg, Germany). A Trolox standard curve was used to quantify FRAP antioxidant activity, and the data were expressed as mg/g Trolox equivalents (TE). Analyses were conducted in triplicate for each replicate.

#### 3.2.10. ABTS Radical Scavenging Activity

The antioxidant activity of the sample was measured by ABTS (2,20-azino-bis-3-ethylbenzo-thiazoline-6-sulphonic acid, Sigma-Aldrich) radical scavenging activity according to Woo et al. (2014) [44]. The assay was designed to quantify the antioxidant activity of the collective phenolic compounds in crude sorghum extracts. Briefly, 50 µL of the sample was mixed with 1 mL of a diluted ABTS solution, left to react for 30 min at room temperature, and then the absorbance was measured at 734 nm wavelength (Thermo Scientific Co., Ltd., USA). ABTS scavenging activity was expressed in mg 100 g^−1^ TE (Trolox equivalent). A Trolox standard curve was used to quantify ABTS radical scavenging activity, and the data were expressed as mg 100 g^−1^ Trolox equivalents (TE). Analyses were conducted in triplicate for each replicate.

#### 3.2.11. UHPLC with Online ABTS

Polyphenol characterisation was conducted using the method by García-Villal et al. (2016) [45] using an Agilent UHPLC (Agilent Technologies, Santa Clara, CA, USA) system with a Zorbax Eclipse Plus C18 column (2.1 mm × 50 mm, 1.8 μm) (Agilent Technologies, CA, USA). The UHPLC system was connected to an autosampler and UV-vis photodiode array detector (PDA). The system was equipped with an exterior binary pump, coil column, and UV-vis detector injecting ABTS solution at a flow rate of 0.6 mL min^−1^. Mobile phase A consisted of deionised water and 0.01% formic acid. Mobile phase B consisted of acetonitrile with 0.01% formic acid. Reconstituted sorghum extracts (20 μL) were injected into the system at an elution gradient of 0–34.98 min, 0–50% A and B; 34.98–37.21 min, 100% B; and 37.31–39.65 min, 100% B. The absorbance was measured at 280 nm for polyphenols, and antioxidant activity of individual polyphenols was determined at 414 nm after passing through the UHPLC system. Trolox was used to quantify the ABTS radical scavenging activity and was expressed as mg 100 g^−1^ Trolox equivalents (TE). Peaks were identified using retention time, peak spectra, and standards. Identified peaks were quantified with their respective standards, and unidentified peaks were quantified as mg 100 g^−1^ GAE [46,47].

#### 3.2.12. Compound Identification Using QTOF LC-MS

Mass spectra of the unknown peaks were determined using the aforementioned UHPLC System connected to an Agilent 6530 Accurate-Mass Q-TOF LC/MS (Agilent Technologies, CA, USA). Peak identification was performed in negative mode; capillary and nozzle voltage was set at 3.5 kV and 500 V, respectively. A complete mass scan ranging from *m*/*z* 50 to 1500 was conducted. Agilent Mass Hunter Qualitative Analysis software version B.07.00 was used to extract time of flight mass spectra, and compounds of the unknown peaks were tentatively identified using the standards listed in 2.1.2 and databases such as ChemSpider (2024) [48], PubChem (2024) [49], Massbank (MassBank, 2006) [50], and Metlin (2006) [51].

#### 3.2.13. Statistical Analysis

Statistical analysis was performed using one-way analysis of variance (ANOVA), followed by post hoc Tukey’s multiple comparisons test using GraphPad Prism 9 software (GraphPad Software Inc., San Diego, CA, USA). The results are reported as mean ± standard deviation. Statistical significance was determined at a level of *p* < 0.05.

## 4. Conclusions

This study demonstrated that processing by cooking with prior fermentation increases the accessibility of bound polyphenols, resulting in an increase in TPC, antioxidant, and radical scavenging activity across the eight varieties investigated. Additionally, the cultivation location (Bellata, NSW) of the white and red pericarp varieties showed a higher number of detectable phenolic compounds. The use of these processing techniques could provide a great number of polyphenols that are readily accessible for human consumption and contain a more diverse range of flavonoids. Despite the many identified phenolic compounds, further investigations are required to determine their bioaccessibility and bioavailability. Furthermore, since some of the compounds identified are yet to be studied for their bioactivity, in vivo dietary intervention studies are warranted to determine the health-promoting effect of processed sorghum foods.

## Figures and Tables

**Table 1:** Impact of processing on the antioxidant activity of sorghum polyphenols.

Variety	Processing Technique	TPC mg/g GAE	TPAC mg/g CE	ABTS mg 100 g^−1^ TE	DPPH mg/g TE	FRAP mg/g TE
BlackSs (Shawaya short black-1)	Raw	9.66 ± 1.66 a	8.38 ± 0.44 a	2.07 ± 0.22 a	11.10 ± 0.06 a	23.27 ± 3.50 a
	Cooked	7.38 ± 0.53 b	4.88 ± 0.67 b	1.47 ± 0.25 b	10.8 ± 0.09 b	28.57 ± 2.22 b
	Fermented	7.93 ± 1.67 c	6.44 ± 0.30 c	1.52 ± 0.08 bc	10.70 ± 0.25 bc	15.80 ± 1.24 c
	Fermented Cooked	10.93 ± 0.85 d	7.05 ± 0.58 c	2.25 ± 0.09 a	11.30 ± 0.06 a	26.81 ± 3.63 ab
BlackSb (Shawaya black)	Raw	6.67 ± 0.64 a	2.01 ± 0.08 a	0.92 ± 0.04 a	2.56 ± 0.14 ab	7.29 ± 1.26 a
	Cooked	5.97 ± 0.61 ab	2.46 ± 0.21 a	1.04 ± 0.12 b	2.76 ± 0.04 c	9.13 ± 1.55 a
	Fermented	6.73 ± 0.77 a	2.45 ± 0.14 a	1.14 ± 0.07 bc	2.61 ± 0.07 ad	11.98 ± 2.00 b
	Fermented Cooked	6.86 ± 0.73 ac	2.65 ± 0.19 b	1.36 ± 0.07 d	2.75 ± 0.08 ce	12.37 ± 1.98 bc
RedBu_1_ (Buster.B)	Raw	0.67 ± 0.08 a	1.31 ± 0.25 a	0.87 ± 0.24 a	2.40 ± 0.19 a	5.09 ± 0.62 a
	Cooked	0.57 ± 0.06 b	1.18 ± 0.18 a	0.91 ± 0.17 a	2.40 ± 0.18 a	5.81 ± 1.18 a
	Fermented	0.52 ± 0.06 bc	1.40 ± 0.33 a	0.86 ± 0.04 a	1.99 ± 0.17 b	9.88 ± 1.08 b
	Fermented Cooked	0.58 ± 0.06 bd	1.44 ± 0.25 a	1.00 ± 0.09 a	2.33 ± 0.12 ac	10.49 ± 0.94 bc
RedBa_1_ (Bazley.B)	Raw	0.50 ± 0.13 a	1.27 ± 0.07 a	0.89 ± 0.06 a	2.29 ± 0.18 a	6.69 ± 0.66 a
	Cooked	0.67 ± 0.25 ac	1.31 ± 0.30 a	1.07 ± 0.20 a	2.61 ± 0.13 b	8.24 ± 1.46 b
	Fermented	0.59 ± 0.05 a	1.24 ± 0.23 a	0.89 ± 0.22 a	1.80 ± 0.17 c	9.72 ± 0.64 c
	Fermented Cooked	0.47 ± 0.10 ab	1.43 ± 0.26 a	0.94 ± 0.11 a	2.15 ± 0.15 a	7.71 ± 1.49 ab
WhiteLi_1_ (Liberty.B)	Raw	0.33 ± 0.01 a	0.05 ± 0.01 a	0.25 ± 0.05 a	0.83 ± 0.19 a	1.55 ± 0.22 a
	Cooked	0.30 ± 0.03 ab	0.12 ± 0.05 b	0.42 ± 0.08 b	1.32 ± 0.21 b	2.40 ± 0.23 bc
	Fermented	0.40 ± 0.04 ac	0.14 ± 0.03 bc	0.46 ± 0.19 bc	0.88 ± 0.21 a	2.79 ± 0.55 bc
	Fermented Cooked	0.34 ± 0.11 a	0.13 ± 0.03 bd	0.54 ± 0.07 b	1.08 ± 0.20 a	2.70 ± 0.50 c
RedBu_2_ (Buster.CC)	Raw	0.58 ± 0.09 ab	0.78 ± 0.05 ab	0.69 ± 0.15 a	1.58 ± 0.18 a	3.74 ± 0.42 a
	Cooked	0.49 ± 0.04 a	0.94 ± 0.10 a	0.84 ± 0.11 a	2.39 ± 0.16 b	6.82 ± 0.57 b
	Fermented	0.42 ± 0.05 ac	1.02 ± 0.16 a	0.73 ± 0.05 a	1.90 ± 0.19 c	5.11 ± 0.91 c
	Fermented Cooked	0.55 ± 0.18 a	1.21 ± 0.46 ac	0.92 ± 0.14 b	1.85 ± 0.19 cd	4.58 ± 0.63 acd
RedBa_2_ (Bazley.CC)	Raw	0.56 ± 0.04 a	1.08 ± 0.28 a	0.84 ± 0.16 a	1.98 ± 0.17 a	5.56 ± 0.40 a
	Cooked	0.45 ± 0.03 b	1.15 ± 0.23 a	0.87 ± 0.10 ab	2.47 ± 0.13 b	7.68 ± 1.22 b
	Fermented	0.43 ± 0.06 a	1.03 ± 0.23 a	0.68 ± 0.08 ac	1.70 ± 0.19 c	4.55 ± 0.72 a
	Fermented Cooked	0.55 ± 0.12 bc	1.52 ± 0.37 b	0.95 ± 0.18 abd	2.08 ± 0.16 a	5.53 ± 0.65 a
WhiteLi_2_ (Liberty.CC)	Raw	0.43 ± 0.02 a	0.04 ± 0.01 a	0.41 ± 0.19 a	0.78 ± 0.22 a	1.07 ± 0.35 a
	Cooked	0.30 ± 0.02 b	0.08 ± 0.02 b	0.47 ± 0.16 a	0.99 ± 0.21 a	1.69 ± 0.32 a
	Fermented	0.29 ± 0.06 bc	0.11 ± 0.02 c	0.46 ± 0.17 a	0.78 ± 0.21 a	2.45 ± 0.77 b
	Fermented Cooked	0.38 ± 0.03 d	0.21 ± 0.04 d	0.46 ± 0.21 a	0.97 ± 0.23 a	2.89 ± 0.65 b

Data are presented as mean ± SD (*n* = 9). Different letters in the different columns represent significant differences at *p* < 0.05. 1, Bellata; 2, Croppa Creek; GAE, gallic acid equivalent; CE, catechin equivalent; TE, Trolox equivalent; TPC, total phenolic content); TPAC, total proanthocyanidins content; ABTS, 2,2′-azino-bis (3-ethylbenzothiazoline-6-sulfonic acid); DPPH, 2,2-diphenyl-1-picrylhydrazyl; FRAP, ferric reducing ability of plasma assay.

**Table 2:** Identification of antioxidant active peaks in sorghum phenolic acetone extracts using *QTOF LC-MS*.

Peak	Tentative ID	Level of ID	m/z	Retention Time	Sorghum Variety	Sorghum Processing Technique			
						Raw	Cooked	Fermented	Fermented Cooked
1	trans-Piceid	2	251.0797	1.06	WhiteLi_1_, RedBa_1_, RedBu_1_, RedBu_2_			**√**	**√**
2	trans cinnamic acid	2	147.0310	1.07	BlackSs				**√**
3	6-methoxy-7-hydroxycoumarin	3	193.0360	1.09	RedBu_1_, BlackSb			**√**	**√**
4	Methyl trans-cinnamic acid	4	161.0479	1.10	WhiteLi_1_, RedBa_2_				**√**
5	Malic acid	2	133.0491	1.11	RedBa_2_, RedBu_2_			**√**	**√**
6	6,8-dimethyl-4-hydroxycoumarin	3	191.0900	1.32	RedBu_1_, RedBu_2_, BlackSb	**√**	**√**		
7	6-prenylnaringenin	4	341.1112	1.70	RedBa_2_, RedBu_1_, RedBu_2_, BlackSs, BlackSb	**√**	**√**		
8	Apigenin-7-*O*-glucoside	3	413.1686	2.16	WhiteLi_2_			**√**	**√**
9	Coumarin	1	145.0510	2.47	RedBa_1_, RedBa_2_, RedBu_2_, BlackSs		**√**	**√**	**√**
10	Protocatechuic acid	1	153.0198	3.00	BlackSs			**√**	**√**
11	Gallic acid	1	170.1200	3.14	WhiteLi_1_				**√**
12	Vanillin	1	153.0188	4.03	BlackSs		**√**		
13	Apigenin-7-*O*-glucoside	3	413.1680	4.39	RedBa_1_			**√**	**√**
14	Quercetin-3,4’-*O*-di-beta-glucopyranoside	4	625.1985	4.50	WhiteLi_1_, WhiteLi_2_, RedBa_1_, RedBa_2_, RedBu_1_, RedBu_2_, BlackSs, BlackSb	**√**	**√**	**√**	**√**
15	Linoleic acid	3	279.1110	4.91	RedBa_1_				**√**
16	Catechin	1	289.0726	5.14	BlackSs				**√**
17	Malvin	5	655.2112	5.47	WhiteLi_1_, WhiteLi_2_, RedBa_1_, RedBa_2_, BlackSs	**√**	**√**		
18	4-methyl-7-aminocoumarin	3	175.0630	6.35	WhiteLi_1_, WhiteLi_2_			**√**	**√**
19	Hippuric acid	1	179.0388	7.30	WhiteLi_1_, WhiteLi_2_, RedBa_1_, RedBa_2_		**√**	**√**	**√**
20	4-Acetylbutyric acid	2	131.0721	7.55	WhiteLi_1_, RedBa_1_, RedBa_2_, RedBu_1_, RedBu_2_, BlackSs, BlackSb			**√**	**√**
21	Caffeic acid	1	181.0512	7.75	WhiteLi_1_, WhiteLi_2_, RedBa_2_, RedBu_1_, RedBu_2_, BlackSs, BlackSb			**√**	**√**
22	3-aminobenzoic acid	2	137.0622	8.55	RedBa_2_, BlackSb			**√**	
23	Procyanidin b1 isomer	4	577.1386	8.97	BlackSs			**√**	**√**
24	Malic acid	2	135.0455	9.03	WhiteLi_1_, WhiteLi_2_, RedBa_1_, RedBa_2_, RedBu_1_, RedBu_2_, BlackSs, BlackSb	**√**	**√**		
25	2-Isopropylmalic acid	3	177.0201	9.20	WhiteLi_1_, WhiteLi_2_, RedBa_1_, RedBa_2_, RedBu_1_, RedBu_2_, BlackSs	**√**	**√**	**√**	**√**
26	Hippuric acid	1	179.0358	9.05	WhiteLi_2_, RedBu_1_, RedBu_2_, BlackSb	**√**	**√**		
27	Catechin	1	289.0733	9.08	BlackSs				**√**
28	Caffeic acid	1	181.0516	9.51	WhiteLi_2_, BlackSs, BlackSb			**√**	**√**
29	Kaempferol-3-*O*-xyloside	3	417.1057	9.66	WhiteLi_2_, RedBa_1_, RedBa_2_, BlackSb	**√**	**√**		
30	Luteolin derivative	3	415.1262	10.05	WhiteLi_1_, WhiteLi_2_, RedBa_1_, RedBu_2_		**√**		
31	Apigeninidin	2	253.0737	10.43	WhiteLi_1_, WhiteLi_2_, RedBa_1_, RedBa_2_, RedBu_1_, RedBu_2_, BlackSs, BlackSb	**√**	**√**	**√**	**√**
32	2-Isopropylmalic acid	2	177.0192	10.91	WhiteLi_2_				**√**
33	Procyanidin C1	4	865.1978	10.45	BlackSs	**√**	**√**	**√**	**√**
34	Epigallocatechin	2	307.1409	11.42	BlackSs				**√**
35	Catechin	1	289.0739	11.46	BlackSs		**√**	**√**	**√**
36	trans cinnamic acid	2	147.0453	11.79	RedBa_1_, RedBu_2_, BlackSs			**√**	**√**
37	trans-Resveratroloside	3	371.1000	12.08	WhiteLi_1_, RedBa_1_, RedBa_2_, RedBu_1_, RedBu_2_, BlackSs, BlackSb	**√**	**√**		
38	Isoferulic acid	2	195.0668	12.09	BlackSb			**√**	**√**
39	2-Hydroxyhippuric acid	2	253.0729	12.11	WhiteLi_2_			**√**	
40	trans-Resveratroloside	3	371.1001	12.16	WhiteLi_2_, BlackSs, BlackSb	**√**	**√**		**√**
41	Catechin derivative	3	720.1572	12.73	BlackSs	**√**	**√**	**√**	**√**
42	N’.n’-dicafferoylspermidine	2	468.2130	13.21	WhiteLi_1_, WhiteLi_2_, RedBa_1_, RedBa_2_, RedBu_1_, RedBu_2_, BlackSs, BlackSb	**√**	**√**	**√**	**√**
43	Ferulic acid	1	468.2156	13.56	WhiteLi_1_, WhiteLi_2_, RedBa_1_, RedBa_2_, RedBu_1_, RedBu_2_, BlackSs, BlackSb	**√**	**√**	**√**	**√**
44	Luteolin	1	285.0736	13.93	BlackSs			**√**	
45	Catechin derivative	3	289.0758	14.21	BlackSs, BlackSb	**√**	**√**		
46	Malic acid	2	135.0458	14.53	WhiteLi_1_, WhiteLi_2_, RedBa_1_, RedBa_2_, RedBu_1_, RedBu_2_, BlackSb			**√**	**√**
47	Catechin	1	289.0738	14.60	BlackSs	**√**			**√**
48	2-Isopropylmalic acid	2	177.0192	14.63	RedBa_2_		**√**		
49	Apigenin	1	269.0475	14.85	BlackSs, BlackSb	**√**	**√**	**√**	
50	Taxifolin-*O*-pentoside	3	399.1681	14.89	RedBa_1_	**√**			
51	Pentahydroxyflavanone-(3 → 4)-catechin-7-*O*-glucoside	4	883.2293	15.00	RedBa_1_, RedBa_2_, RedBu_1_, RedBu_2_, BlackSs, BlackSb	**√**	**√**	**√**	**√**
52	Eriodictyol deoxyhexoside	3	433.1181	15.12	RedBa_1_, RedBa_2_, RedBu_1_, RedBu_2_	**√**	**√**		
53	Eriodictyol deoxyhexoside	3	433.1169	15.45	RedBu_1_, RedBu_2_, BlackSs		**√**	**√**	**√**
54	Glucomalcomiin	2	482.2334	15.39	RedBa_1_, RedBu_1_, BlackSs, BlackSb	**√**		**√**	**√**
55	Procyanidin C1	4	865.2171	15.43	RedBa_2_, RedBu_2_, BlackSs	**√**		**√**	
56	3-aminobenzoic acid	2	137.0613	15.45	WhiteLi_1_, RedBu_1_			**√**	**√**
57	Kaempferol	2	187.0980	15.67	WhiteLi_1_, WhiteLi_2_, RedBa_1_, RedBa_2_, RedBu_2_, BlackSs, BlackSb	**√**	**√**	**√**	**√**
58	Epicat-(4beta → 6)-epicatechin-(2beta- > 7,4beta → 8)-epicatechin	2	867.2377	16.36	RedBu_1_			**√**	**√**
59	Procyanidin C1	4	867.2382	16.37	RedBa_1_, RedBa_2_, RedBu_1_, RedBu_2_, BlackSs	**√**	**√**	**√**	**√**
60	Procyanidin b1 isomer	4	577.1590	16.79	WhiteLi_1_, WhiteLi_2_	**√**			
61	Luteolin-7-*O*-glucoside	2	447.0954	16.81	RedBa_1_, RedBa_2_, RedBu_1_, RedBu_2_	**√**	**√**		**√**
62	Pyrano-eriodictyol-(3 → 4)-catechin-7-*O*-glucoside	4	867.2362	16.99	RedBa_1_, RedBa_2_, RedBu_1_, BlackSs	**√**	**√**	**√**	**√**
63	Apigenin	1	271.0632	17.05	RedBa_1_, BlackSs, BlackSb	**√**	**√**		
64	Taxifolin	2	303.0527	17.69	RedBa_1_, RedBu_1_, BlackSs, BlackSb	**√**		**√**	**√**
65	Malvidin-3-*O*-glucoside	2	329.2133	17.91	RedBa_1_				**√**
66	Procyanidin	2	429.2133	17.98	RedBa_1_, RedBa_2_, RedBu_1_, RedBu_2_, BlackSb	**√**	**√**	**√**	**√**
67	Pyrano-naringenin-(3 → 4)-catechin-7-*O*-glucoside isomer	3	851.2415	18.55	RedBa_1_, RedBa_2_, RedBu_1_, RedBu_2_, BlackSs	**√**	**√**	**√**	**√**
68	Taxifolin	2	303.0879	19.23	WhiteLi_1_, WhiteLi_2_, RedBa_1_, RedBa_2_, RedBu_1_, RedBu_2_, BlackSs, BlackSb	**√**	**√**	**√**	**√**
69	Kaempferol	2	187.0984	19.32	WhiteLi_2_				**√**
70	Luteolin derivative	3	415.1061	19.35	BlackSb	**√**			
71	Taxifolin-*O*-pentoside	2	399.1102	19.75	BlackSb	**√**			
72	Quercetin-3-*O*-glucuronide	2	478.9223	19.84	WhiteLi_1_, WhiteLi_2_	**√**			
73	Eriodictyol deoxyhexoside	3	721.1826	19.98	RedBa_1_		**√**		
74	Pentahydroxyflavanone-(3 → 4)-catechin-7-*O*-glucoside isomer	4	721.1780	19.99	RedBa_1_, RedBa_2_, RedBu_1_, RedBu_2_	**√**	**√**	**√**	**√**
75	Eriodictyol deoxyhexoside	3	433.1121	20.61	RedBa_2_		**√**		
76	Luteolin	1	285.0766	21.45	RedBa_1_, RedBa_2_, RedBu_1_, RedBu_2_, BlackSs, BlackSb	**√**	**√**	**√**	**√**
77	7-*O*-methyl-luteolinidin	5	395.2135	21.49	WhiteLi_1_, WhiteLi_2_, RedBa_1_, RedBa_2_, RedBu_1_, RedBu_2_, BlackSs	**√**	**√**	**√**	**√**
78	Voacamine	2	705.1871	21.83	RedBa_1_, RedBa_2_, RedBu_2_	**√**		**√**	**√**
79	Procyanidin	2	429.1216	22.12	BlackSb	**√**			
80	Luteolin derivative	3	415.1070	22.41	WhiteLi_1_, WhiteLi_2_, RedBa_1_, RedBa_2_, RedBu_1_, RedBu_2_, BlackSs, BlackSb	**√**	**√**	**√**	**√**
81	Taxifolin-*O*-pentoside	3	399.1104	24.36	WhiteLi_1_, WhiteLi_2_, RedBa_1_, RedBa_2_, RedBu_1_, RedBu_2_, BlackSs, BlackSb	**√**	**√**		**√**
82	Apigenin	1	271.0627	24.43	RedBu_1_			**√**	
83	Procyanidin	2	429.1212	24.91	WhiteLi_1_, WhiteLi_2_, RedBa_1_, RedBa_2_, RedBu_1_, RedBu_2_, BlackSs, BlackSb	**√**	**√**		
84	Apigenin	1	269.0471	25.17	RedBa_1_		**√**		
85	Gallic acid hexoside	3	331.2498	25.92	RedBa_2_, RedBu_1_, RedBu_2_, BlackSs	**√**		**√**	
86	Malvidin-3-*O*-glucoside	5	329.2539	25.93	RedBa_1_, RedBu_1_		**√**	**√**	
87	Apigenin-7-*O*-glucoside	2	413.1276	27.05	BlackSb	**√**			

## Data Availability

The original contributions presented in the study are included in the article, further inquiries can be directed to the corresponding authors.

## Abbreviations

ABTS: 2:2′-azino-bis (3-ethylbenzothiazoline-6-sulfonic acid); C: cooked; F: fermented; FRAP: ferric reducing antioxidant power; FC: fermented cooked; GAE: gallic acid equivalent; QTOF LC-MS: quadrupole time of flight liquid chromatography mass spectra; R: raw; TE: Trolox equivalent; TPC: total phenolic content; TPTZ: tris(2-pyridyl)-s-triazine; UHPLC: ultra-high-performance liquid chromatography.
